# An N-Rich Polymer for the Selective Recovery of Gold from Wastewater

**DOI:** 10.3390/molecules29102398

**Published:** 2024-05-20

**Authors:** Haonan Dong, Ge Shang, Yi Zhang, Enrui Dai, Mingdong Shao, Chunfeng Chen, Hongxing He, Zhifeng Nie, Mingyang Xiong, Deren Miao, Sibiao Zhao

**Affiliations:** 1 Yunnan Key Laboratory of Metal-Organic Molecular Materials and Device, School of Chemistry and Chemical Engineering, Kunming University, Kunming 650214, China; 2 Kunming Institute for Food and Drug Control, Kunming 650034, China

**Keywords:** gold resource recovery, selective adsorption, adsorption mechanism, adsorptiological modeling, DFT calculations

## Abstract

The recovery of valuable gold from wastewater is of great interest because of the widespread use of the precious metal in various fields and the pollution generated by gold-containing wastes in water. In this paper, a water-insoluble cross-linked adsorbent material (TE) based on cyanuric chloride (TCT) and ethylenediamine (EDA) was designed and used for the adsorption of Au(III) from wastewater. It was found that TE showed extremely high selectivity (D = 49,213.46) and adsorption capacity (256.19 mg/g) for Au(III) under acidic conditions. The adsorption rate remained above 90% eVen after five adsorption–desorption cycles. The adsorption process followed the pseudo-first-order kinetic model and the Freundlich isotherm model, suggesting that physical adsorption with a multilayer molecular overlay dominates. Meanwhile, the adsorption mechanism was obtained by DFT calculation and XPS analysis, and the adsorption mechanism was mainly the electrostatic interaction and electron transfer between the protonated N atoms in the adsorbent (TE) and AuCl_4_^−^, which resulted in the redox reaction. The whole adsorption process was the result of the simultaneous action of physical and chemical adsorption. In conclusion, the adsorbent material TE shows great potential for gold adsorption and recovery.

## 1. Introduction

Precious metal elements are commonly used in the chemical industry because of their favorable physical and chemical properties [1]. Gold, a precious metal element, has been used for many years as jewelry and currency [2]. Additionally, it plays a crucial role in high-tech fields such as catalysis [3] and electronics [4]. In recent years, there has been an increasing demand for gold in industry, but the amount of gold ore in nature has continuously decreased because of heavy mining. This has led to an increase in the price of gold [5]. Additionally, the accelerated iteration of current electronic products has generated a large amount of electronic waste, which contains a significant amount of gold elements. This phenomenon can cause pollution and environmental damage [6]. Therefore, the recovery and treatment of secondary gold resources are of great significance.

Various methods have been developed for gold recovery, including precipitation [7], replacement [8], solvent extraction/reverse extraction [9], and adsorption [10]. Among these, adsorption is a commonly used and advantageous method because of its simple synthesis of adsorbent materials, ease of operation, and low cost. Numerous adsorbent materials, such as resin [11,12], covalent organic frameworks (COFs) [13,14], metal–organic frameworks (MOFs) [15,16,17], and polymers [18,19], have been employed for adsorption. Polymers are commonly used as adsorbent materials for gold recovery. For instance, Chen et al. [20] developed cationic pyridine polymers with different configurations (4-AP/PCMS) to selectively recover gold from WPCB leachate. The maximum adsorption capacity was found to be 437.68 mg/g, and a high adsorption rate was maintained even after five cycles of adsorption and desorption. In a study by Wang et al. [21], polyaniline was modified with trimethyl phosphate for the selective adsorption of gold from aqueous waste solutions. The maximum adsorption capacity was found to be 881 mg/g, and the presence of interfering ions did not affect the adsorption of gold in actual wastewater applications, indicating good selectivity of the adsorbent. In conclusion, it is evident that polymers exhibit exceptional performance in the adsorption and recovery of gold.

The use of inexpensive raw materials and simple methods in the recovery of gold from wastewater is cost-effective and far-reaching. Ethylenediamine (EDA) is a low-cost compound rich in amine group N, which has been used excellently in the modification of polymers. It has been shown that N-containing compounds increase the binding sites on the adsorbent surface [22]. Cyanuric chloride is rich in N elements and rigid rings. The use of TCT for cross-linking with EDA, on the one hand, can overcome its disadvantage of being difficult to recover from water. On the other hand, it is more favorable for adsorption of metal chloride anions [23]. Therefore, in this paper, we synthesized a water-insoluble cross-linked polymeric adsorbent (TE) through a substitution reaction between EDA and TCT for trapping Au(III) in wastewater. In this method, the protonated N-rich adsorbent (TE) exhibits excellent selectivity for Au(III) in mixed ionic solutions because of its positive charge. It also shows good performance in recycling. Furthermore, the combination of adsorption experiments and DFT calculations systematically explains the occurrence of the adsorption process.

## 2. Results

### 2.1. Characterization and Chemical Properties

**Figure 1:**
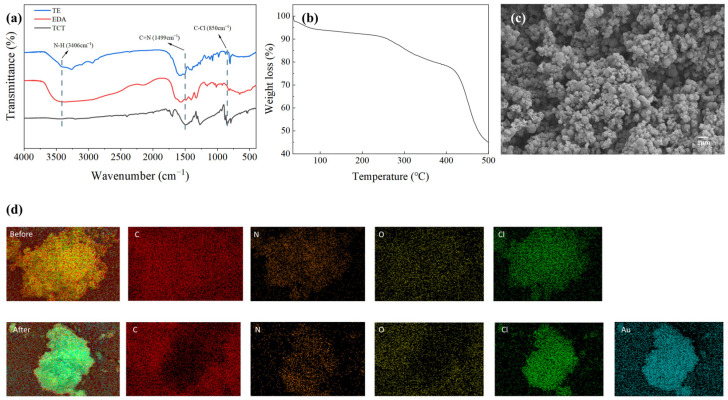
Characterization of TE materials (**a**) FTIR; (**b**) TG; (**c**) SEM; and (**d**) mapping.

TE and raw material EDA and TCT were characterized by FTIR spectra. The spectra (Figure 1a) show a characteristic peak of the C=N backbone vibration on the triazine ring at 1499 cm^−1^ [24]. Additionally, the characteristic peak at 3406 cm^−1^ corresponds to the N-H stretching vibration, and the peak at 2940 cm^−1^ corresponds to the C-H vibration. The peak at 850 cm^−1^ is characteristic of the stretching vibration of the C-Cl bond [25]. In the infrared spectrum of TE, the intensity of this peak almost disappeared because of the cross-linking of TCT with EDA, indicating the successful synthesis of TE adsorbent materials.

The TE adsorbent materials were synthesized and subjected to thermogravimetric analysis (TGA). Figure 1b shows that the weight loss of the adsorbent materials up to 100 °C is mainly due to the loss of free water in the samples, which is approximately 6.1%. Above 250 °C, the small portion of the synthesized polymer that has a low degree of polymerization slowly decomposes because of the high temperature. After 400 °C, some of the polymers with a high degree of polymerization also start to decompose rapidly. These results indicate that TE adsorbent materials are thermally stable up to 250 °C. The results show that TE adsorbent materials have good thermal stability.

**Figure 2:**
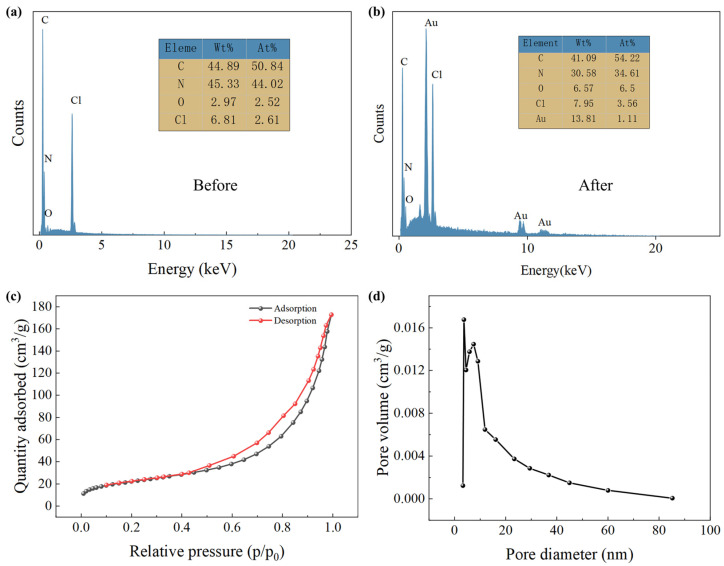
(**a**,**b**) EDS elemental spectra; (**c**) BET adsorption–desorption isotherms; and (**d**) pore size distribution.

The morphology of TE was analyzed using scanning electron microscopy (SEM) (Figure 1c). The results show that the particle size of TE is mainly distributed within 1 μm. The surface of the sample is clustered with granular spheres of different sizes, with obvious voids between the particles, which is conducive to the adsorption process. The EDS analysis provides information on the elemental content of the material’s surface before and after adsorption, as well as the changes in elemental content. This information is presented through EDS elemental spectra (Figure 2a,b) and mapping (Figure 1d) analysis. Figure 1b shows that the content of Au after adsorption is 1.11%. The adsorbed chloroauric acid solution contains a certain amount of elemental Cl, resulting in an increase in the content of elemental Cl on the material’s surface after adsorption. It can be concluded that the synthesized TE adsorbent has a good adsorption capacity for Au(III).

To assess the adsorption capacity of the TE adsorbent, which is mainly determined by the number of ions in contact with the adsorbent material, the pore size distribution was examined through N_2_ adsorption–desorption isotherms. The BET test curves (Figure 2c) indicate a slow climb in the low P/P_0_ region, followed by a rapid increase in the higher P/P_0_ region, consistent with type IV. Table 1 demonstrates the remaining parameters. The pore size distribution is shown in Figure 2d, which indicates that the pore size is mainly distributed in the region below 50 nm. TE’s specific surface area is 80.1599 m^2^/g, with a porosity of 0.262001 cm^3^/g and an average pore size of 11.3548 nm. These results suggest that TE has multiple reaction sites and ample space for the adsorption of Au(III).

### 2.2. Adsorption Properties

#### 2.2.1. Effect of pH on Adsorption Experiments

**Figure 3:**
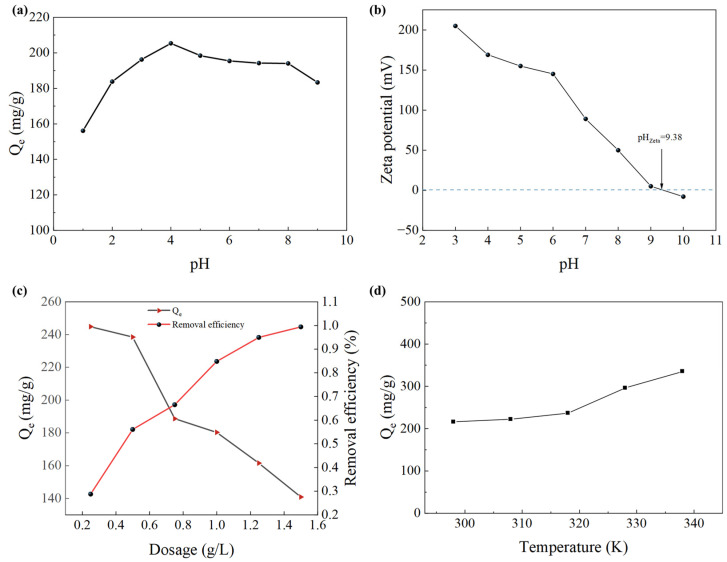
(**a**) Effect of pH on the experiment. (**b**) Zeta potential plot. (**c**) Effect of TE dosage. (**d**) Effect of temperature on the experiment.

It is important to note that the adsorption effect of the adsorbent is influenced by the pH of the solution. The experimental conditions were optimized by investigating the adsorption capacity of TE on 100 mg/L of Au(III) at pH values ranging from 1 to 9. Figure 3a demonstrates that the adsorption capacity increased as the pH decreased below 4 and decreased as the pH exceeded 4. This suggests that the optimal adsorption effect of TE on Au(III) occurred at pH 4. Furthermore, this study analyzed the zeta potential of PE at different pH levels (Figure 3b). The results indicate that the surface of the adsorbent carries a positive charge when the pH is below 9.38. This positive charge facilitates electrostatic interaction with negatively charged AuCl_4_^−^, thereby promoting the adsorption process.

#### 2.2.2. Effect of Adsorbent Dosage

The impact of varying the TE dosage on the adsorption capacity and removal efficiency was examined. The results of the experiments are presented in Figure 3c. As the dosage of the TE increased from 0.25 g/L to 1.5 g/L, the removal efficiency exhibited a gradual increase, while the Q_e_ exhibited a gradual decrease. Finally, the removal efficiency reached a value close to 100% at a TE dosage of 1.5 g/L. The observed increase in removal efficiency may be attributed to the fact that the increase in TE dosage resulted in an increase in the number of binding sites. Conversely, the excessive use of adsorbent will result in the creation of more vacant binding sites, resulting in a decrease in adsorption capacity [26]. Considering the effects of the above two points, the adsorbent (TE) dosage was selected as 0.5 g/L.

#### 2.2.3. Thermodynamic Studies

Considering the effect of temperature on the adsorption capacity at the time of adsorption, temperatures of 298 K, 308 K, 318 K, 328 K, and 338 K were selected for the adsorption of Au(III) at pH 4 for 1 h. The results of the experiments are shown in Figure 3d. The thermodynamically relevant values of ΔG, ΔH, and ΔS were calculated using the Gibbs free energy equation. The value of ΔG was calculated using Equation (1), while the values of ΔH and ΔS were obtained from the slope and intercept of [27] lnKc versus 1/T in Equation (2). The thermodynamic parameters are shown in Table 2.

(1) $$\Delta G=-RTln(1000K_{c})$$

(2) $$lnK_{c}=-\frac{\Delta H}{RT}+\frac{\Delta S}{R}$$

where R is the universal gas constant (8.314 J/mol·K), T is the absolute temperature (K), and K_c_ is the equilibrium constant (K_c_ = Q_e_/C_e_). Q_e_ (mg/g) is the adsorbed amount of Au(III) ions at equilibrium and C_e_ (mg/L) is the equilibrium concentration of Au(III) in solution.

The ΔG value decreases as the temperature increases, suggesting that adsorption is more likely to occur at higher temperatures. Meanwhile, a negative value of ΔG indicates that the adsorption process is spontaneous. A positive value of ΔH indicates that the adsorption process is an endothermic reaction and increasing the temperature can make the reaction move forward, which is favorable for adsorption. A positive value of ΔS indicates that the adsorption process is an entropy-increasing process and the system changes from the ordered state to the disordered state. The ions in the solution may move freely during the adsorption process, increasing the disorder in the system. Therefore, the adsorption process is a spontaneous process that involves heat absorption and an increase in entropy.

#### 2.2.4. Kinetic Studies

The impact of adsorption time on the adsorption of Au(III) was investigated at pH 4, 298 K, and an initial concentration of 100 mg/L. The adsorption process was carried out using TE. The results indicated that TE reached adsorption saturation with 1 h, with a saturation adsorption capacity of 191.55 mg/g. A more detailed investigation into the adsorption kinetics between the adsorbent and Au(III) during the adsorption process was conducted. The pseudo-first-order kinetic model of Equation (3) and the pseudo-second-order kinetic model of Equation (4) were used to non-linearly fit the data [28].

(3) $$Q_{t}=Q_{e}\left(1-e^{-k_{1}t}\right)$$

(4) $$Q_{t}=\frac{{Q_{e}}^{2}k_{2}t}{1+Q_{e}k_{2}t}$$

where Q_t_ (mg/g) and Q_e_ (mg/g) are the adsorption capacity of Au(III) at time t (min) and equilibrium, respectively, k_1_ is the rate constant for the pseudo-first-order kinetic model, and k_2_ is the rate constant for the pseudo-second-order kinetic model at equilibrium.

**Figure 4:**
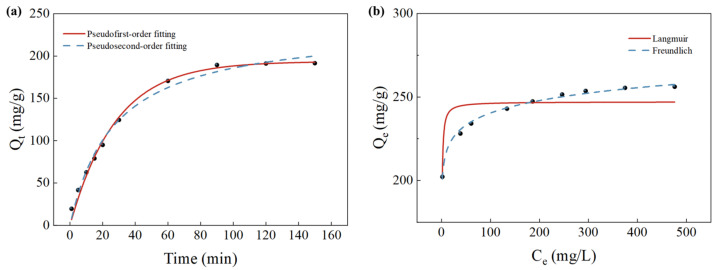
(**a**) Pseudo-first-order and pseudo-second-order kinetic fit curves. (**b**) Langmuir isothermal and Freundlich isothermal model fit curves.

As demonstrated in Figure 4a and Table 3, the correlation coefficient of the fitted pseudo-first-order kinetic curves (R_1_^2^ = 0.991) is greater than that of the pseudo-second-order kinetic curves (R_2_^2^ = 0.988). This indicates that the adsorption process can be accurately represented by the pseudo-second-order kinetic model, which suggests that the adsorption process is dominated by chemisorption.

#### 2.2.5. Adsorption Isotherms

To investigate the adsorption behavior during the process, the effect of varying initial Au(III) concentrations on adsorption capacity was studied at 298 K, pH 4, and an adsorption time of 1 h. As shown in Figure 4b, the adsorption capacity increased gradually with an increase in the initial concentration of Au(III). After the initial concentration reached 300 mg/L, the adsorption capacity tended to stabilize. To further investigate the adsorption mechanism, we used the Langmuir isothermal model (Equation (5)) and the Freundlich isothermal model (Equation (6)) to fit the experimental data [29].

(5) $$Q_{e}=\frac{Q_{m}k_{L}C_{e}}{1+k_{L}C_{e}}$$

(6) $$Q_{e}=k_{F}{C_{e}}^{1/n}$$

where Q_e_ (mg/g) is the adsorption capacity of Au(III) ions at equilibrium, C_e_ (mg/L) is the equilibrium concentration of Au(III) ions in solution, Q_m_ (mg/g) is the maximum adsorption capacity of Au(III) ions, and k_L_ is a constant in the Langmuir model. k_F_ and n are constants in the Freundlich model.

The correlation parameters obtained from fitting the Langmuir and Freundlich isothermal model curves are listed in Table 4. The Langmuir isothermal model correlation coefficient was R_1_^2^ = 0.699, while the Freundlich isothermal model correlation coefficient was R_2_^2^ = 0.993. These results suggest that the adsorption process is more in line with the Langmuir isotherm model and belongs to physical adsorption with multilayer coverage.

### 2.3. Selectivity and Recycling Studies

**Figure 5:**
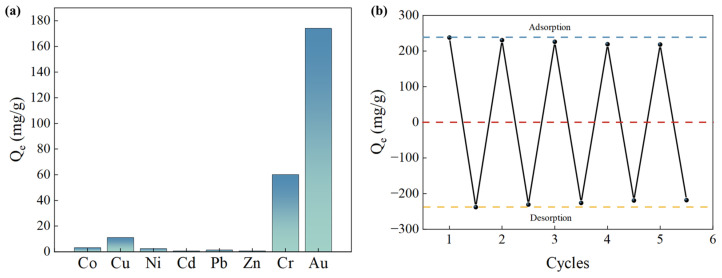
(**a**) Selectivity of the adsorbent. (**b**) Line graph of the number of cycles.

To investigate the adsorbent’s selectivity to ions, we selected metal ions including Co, Cu, Ni, Cd, Pb, Zn, and Cr as interfering ions. The metal ions were adsorbed with TE as the adsorbent for 1 h at 298 K and pH 4, with an initial concentration of 100 mg/L. The results (Figure 5a) indicate that the adsorbent’s capacity for Au(III) is significantly greater than for the other metal ions. The adsorbent also has an adsorption effect on Cr ions, possibly because both Cr ions and Au ions carry a negative charge under acidic conditions. Table 5 shows the results of simultaneous calculations of the distribution ratio (D) and the selectivity coefficient (K).

In the selectivity experiments, it is evident that the adsorbent has a much higher distribution ratio (D) for Au(III) ions compared with several of the other metal ions. This result is due to the electrostatic repulsion between the positive chargeability of the other metal ions and the protonated TE, which is not conducive to adsorption behavior. The selectivity coefficient (K) represents the proximity of the distribution ratios of the two metal ions, and a smaller value indicates that the adsorption of the adsorbent on the two metal ions is more similar. This is consistent with the results of the distribution ratio (D) mentioned above. In summary, the adsorbent demonstrates superior specificity and selectivity for the adsorption of Au(III).

To investigate the cycling performance of TE, we carried out the desorption process on TE after adsorbing Au(III) using a mixture of 0.5 M thiourea and hydrochloric acid. This was performed by adding the above mixture to the adsorbed TE for ultrasonication, shaking for 10 min, removing the mixture, and then repeating the above operation three times. As shown in Figure 5b, TE maintained over 90% of adsorption after five adsorption–desorption processes, indicating that the synthesized adsorbent material (TE) has good cyclability.

### 2.4. Mechanism Studies

**Figure 6:**
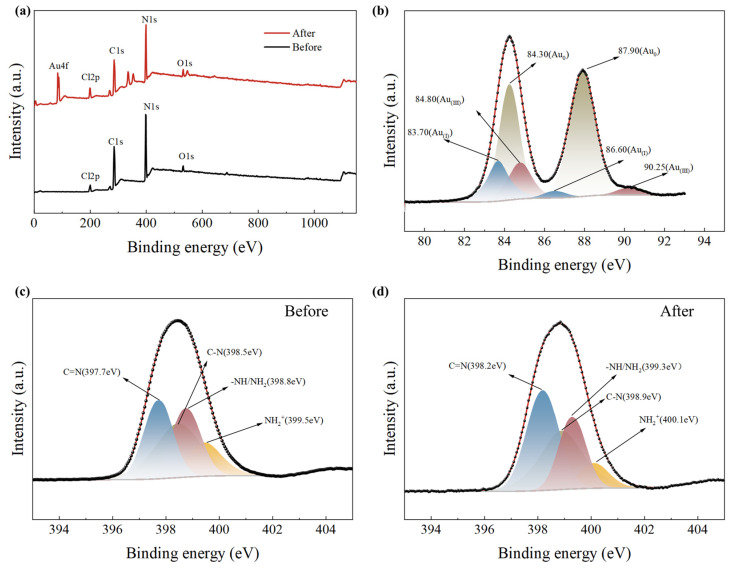
(**a**) Total XPS spectra before and after adsorption; (**b**) Au4f spectra after adsorption; (**c**,**d**) N1s spectra before and after adsorption.

To investigate the adsorption mechanism, we analyzed the binding sites of the adsorbent materials before and after adsorption using XPS. The total spectra before and after adsorption show that the characteristic peaks of Au appeared in the spectra after adsorption, indicating successful adsorption of Au by the adsorbent (Figure 6a). Comparison of the N1s spectra before and after adsorption reveals that the peaks corresponding to the positions of 398.2 eV, 398.9 eV, 399.3 eV, and 400.1 eV are shifted after adsorption compared with those before adsorption. Specifically, the peaks corresponding to C=N (397.7 eV), C-N (398.5 eV), -NH/NH_2_ (398.8 eV), and -NH_2_^+^ (399.5 eV) shifted to the high energy region [30] (Figure 6c,d). This shift is attributed to the oxidation of the adsorbent by the adsorbed gold ions, indicating that the adsorption process is accompanied by a charge transfer phenomenon. Observation of the Au4f spectra after adsorption shows that the gold element exists in the following three states: Au0 (4f_7/2_ at 84.30 eV and 4f_5/2_ at 87.90 eV), Au(I) (4f_7/2_ at 83.70 eV and 4f_5/2_ at 86.60 eV), and Au(III) (4f_7/2_ at 84.80 eV and 4f_5/2_ at 90.25 eV) [31] (Figure 6b). It can be seen that there are lower valence states of Au. This is due to the adsorption process where Au(III) is adsorbed by the adsorbent and then undergoes a reduction reaction, thus being reduced to a lower valence state. Overall it can be concluded that chemisorption occurs between the adsorbent and Au(III) during the process of adsorption.

**Scheme 1:**
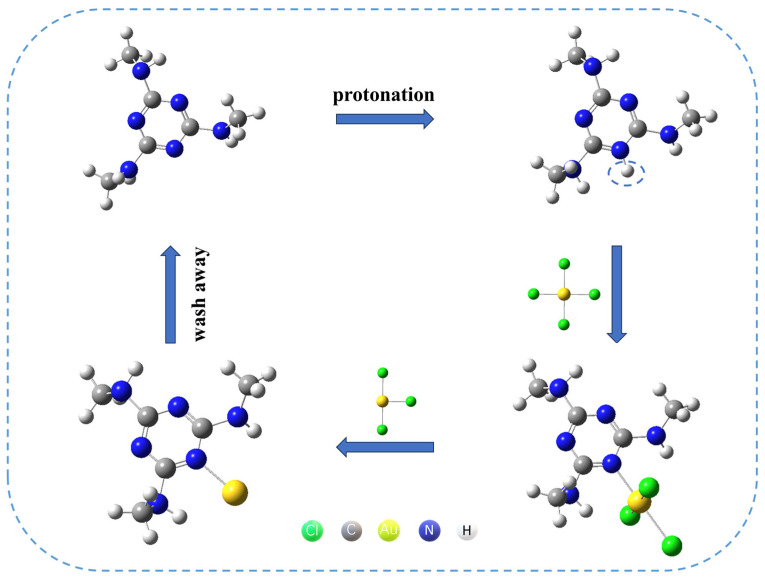
The mechanism of adsorption of AuCl_4_^−^ by the adsorbent (TE).

The adsorption mechanism of AuCl_4_^−^ by the adsorbent (TE) is shown in Scheme 1, starting with the protonation of the N atom on TE. Then, the protonated TE and AuCl_4_^−^ produce the intermediate substance AuCl_3_, after which a redox reaction reduces Au(III) to Au(0). Finally, the adsorbent is eluted and reused.

The validation of TE’s adsorption sites was carried out using the Fukui function, which was calculated by DFT. The values of f_(r)_^+^ and f_(r)_^−^ for TE were calculated using Equations (7) and (8) [32], respectively. These values indicate the extent to which the regions are attacked nucleophilically and electrophilically. Table 6 shows the results of the Fukui function of TE (list of representative sections).

(7) $$f_{\left(r\right)}^{+}=q_{N}^{r}-q_{N+1}^{r}\;$$

(8) $$f_{\left(r\right)}^{-}=q_{N-1}^{r}-q_{N}^{r}\;$$

where $q_{N}^{r}$ represents that the adsorbent has not gained or lost an electron, $q_{N+1}^{r}$ represents that the adsorbent has gained an e^−^, and $q_{N-1}^{r}$ represents that the adsorbent has lost an e^−^.

**Figure 7:**
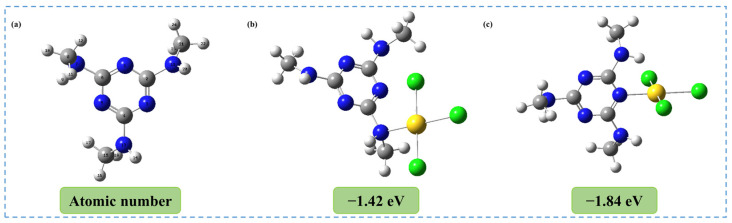
(**a**) Atomic number of TE; (**b**,**c**) Binding energy between the protonated TE and Au(III).

The f_(r)_^+^ values indicate that N3 is more susceptible to the negatively charged AuCl_4_^−^ attack, while N19 is relatively weaker. The f_(r)_^−^ values indicate that N19 is more susceptible to electrophilic attack by H+ and thus protonation, while N3 is relatively more difficult to protonate. Therefore, the adsorption process involves electrostatic adsorption between protonated N19 and AuCl_4_^−^, followed by chemical bonding between protonated N3 and AuCl_4_^−^. This result also confirms the results obtained from the previous experimental fitting, where the adsorption process was mainly dominated by physical adsorption, while chemical adsorption was relatively weak. Then the binding energies of N protonated at different positions with AuCl_3_ were calculated as −1.84 eV for N3 protonation and −1.42 eV for N19 protonation (Figure 7a–c). Based on the results, it can be concluded that the N on the triazine ring can bind better to Au(III), which is in agreement with the results of Fukui function.

**Figure 8:**
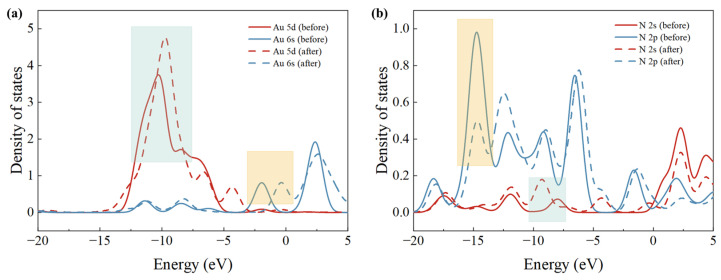
Partial density of states (PDOS) of (**a**) Au and (**b**) N atoms.

In order to study the electron transfer process during adsorption, partial density of states (PDOS) calculations were carried out using Multiwfn software [33]. In Figure 8a, it can be seen that the 5d orbital of Au is significantly enhanced after adsorption near −10 eV, and the 6s orbital is significantly shifted around 0 eV. Figure 8b shows the 2s and 2p orbitals of N. It can be seen that the 2s orbital of N is shifted considerably near −10 eV, while the 2p orbital is substantially weakened after adsorption near −15 eV. The above phenomena indicate that the adsorption process of TE with Au(III) is accompanied by the transfer of electrons, suggesting that there is chemisorption in the adsorption process. This is consistent with previous XPS analyses. In summary, the whole adsorption process is accompanied by the combined effect of physical and chemical adsorption.

## 3. Materials and Methods

### 3.1. Materials and Instruments

The instruments used in this study included a pH meter (PHS-3C, INESA, Shanghai, China), a Fourier Transform Infrared Spectrometer (Cary-6400, Agilent, Santa Clara, CA, USA), a Scanning Electron Microscope (S4800, Hitachi, Tokyo, Japan), Thermal Stability Analyser (6300, Hitachi, Tokyo, Japan), an X-ray Photoelectron Spectrometer (Escalab 250 Xi, Thermo Scientific, Waltham, MA, USA), and a coupled plasma atomic emitter (Icap-6300, Thermo Scientific, Waltham, MA, USA).

TCT (99%) was purchased from Shanghai McLean Biochemical Technology Co., Ltd.; EDA, tetrahydrofuran (THF, 99%), and potassium dichromate were provided by Chengdu Cologne Chemical Co., Ltd. (Chengdu, China); HAuCl_4_·4H_2_O was purchased from Sinopharm Chemical Reagent Co., Ltd. (Shanghai, China); and other metal salt solutions purchased fromTianjin Fengchuan Chemical Reagent Technology Co., Ltd. (Tianjin, China). All reagents were used as received without further purification. The experimental water was ultrapure water (18.25 MΩ).

### 3.2. Preparation of TE

The selection of TCT and EDA as reactants was mainly based on the consideration that the N atoms on the rigid triazine ring of TCT could easily react with Au(III) to produce an adsorption effect, but its slightly water-soluble nature could affect the adsorption effect. Therefore, EDA was selected as the cross-linking agent, and the two reacted to produce a water-insoluble polymeric adsorbent (TE). A specific procedure for synthesizing TE was carried out as follows: TCT (1.0 g, 5.42 mmol) and 50.0 mL of tetrahydrofuran (THF) were added to a three-necked flask. The mixture was stirred at 500 rpm until it became homogeneous. EDA (0.978 g, 16.2 mmol) was then slowly added dropwise to the three-necked flask. After completing the addition, the mixture was reacted at 60 °C for 36 h. Finally, the suspension was placed in an ice-water bath to precipitate. The solid was washed three times with tetrahydrofuran and then with water several times before being freeze-dried to obtain a water-insoluble cross-linked ethylenediamine (TE) white powder (1.19 g).

### 3.3. Adsorption Experiments

Batch adsorption experiments were conducted using 10 mg of TE as an adsorbent in 20 mL of a Au(III) solution with varying concentrations. The mixtures were shaken in a shaker at 25 ± 1 °C for a defined time, and the concentration of metal ions was determined by ICP-AES. The experiments were conducted in triplicate, and the results were averaged to reduce experimental error. The theoretical adsorption was calculated using Equations (9) and (10) [34].

(9) $$Q_{e}=\frac{C_{0}-C_{e}}{1000W}\times V$$

(10) $$Q_{t}=\frac{C_{0}-C_{t}}{1000W}\times V$$

where Q_e_ (mg/g) is the theoretical adsorption capacity; C_0_ (mg/L), C_e_ (mg/L), and C_t_ (mg/L) are the initial, equilibrium, and moment t concentrations of the metal ions, respectively; W (g) is the mass of the adsorbent used; and V (mL) is the volume of the solution.

Adsorption experiments were conducted in a water bath thermostatic oscillator at 298 K. The experiment examined the effect of pH by placing 10 mg of adsorbent into 20 mL of 100 mg/L Au(III) solution with varying pH levels (1–9). Similarly, 10 mg of adsorbent was added to 20 mL of a 100 mg/L Au(III) solution at the same pH. The adsorption experiments were conducted in a water bath thermostatic oscillator at 298 K. Samples were taken at different time points (1, 5, 10, 15, 20, 30, 60, 90, and 120 min) to examine the effect of time on the experiments. A total of 10 mg of adsorbent was added to 20 mL of a 200 mg/L Au(III) solution at the same pH for 1 h. Adsorption experiments were conducted at different temperatures (298 K, 308 K, 318 K, 328 K, and 338 K) to investigate the impact of temperature on the experimental adsorption amount. A total of 10 mg of adsorbent was added to various concentrations of Au(III) solutions (100, 150, 200, 250, 300, 350, 400, 500, and 600 mg/L) at the same pH and temperature (298 K). After 1 h of adsorption, the samples were taken to examine the effect of the initial concentration of Au(III) on the adsorption experiments. The concentration of Au(III) was determined before and after adsorption using ICP-AES.

The selectivity experiments considered Co, Cu, Ni, Cd, Pb, Zn, and Cr metal ions. The experiments were conducted at 298 K and pH 4, where 10 mg of adsorbent was added to a 20 mL mixed solution containing other metal ions and Au(III). The concentration of ions before and after adsorption was determined by ICP-AES. Equations (11) and (12) were used to calculate the partition ratio (D) and the selectivity coefficient (K), respectively [35].

(11) $$D=\frac{C_{0}-C_{e}}{C_{e}}\times \frac{V}{W}$$

(12) $$K=\frac{D_{Au}}{D_{M}}$$

where C_0_ (mg/L) and C_e_ (mg/L) are the initial and equilibrium concentrations of the metal ions, respectively. V (mL) is the volume of the metal ion solution, W (g) is the mass of the adsorbent, and M represents the other competing metal ions.

### 3.4. DFT Calculations

This paper presents Density Functional Theory (DFT) calculations using Gaussian 16W. Repeated structural units of TE were selected as substitutes. BP86 (solvent = water, em = d3)/def2svp was used for structure optimization, while the single point energy was calculated using B2PLYP/def2tzvpp [15]. The Fukui function was used to calculate the binding sites of TE. Equation (13) below was used to calculate the adsorption energy between the adsorbent and the metal ion [36].

(13) $$E_{\left(ad\right)}=E_{\left(total\right)}-E_{\left(TE\right)}-E_{\left(Au\right)}$$

where E_total_, E_TE_, and E_Au_ are the energies of the adsorbed complex, adsorbent TE, and adsorbed Au(III), respectively.

## 4. Conclusions

This work presents the generation of a polymeric adsorbent (TE) through cross-linking between EDA and TCT. TE was characterized using FTIR, SEM, EDS, and BET, which indicated that the material has good adsorption properties. The adsorption process conditions were optimized by batch adsorption experiments. Kinetic, thermodynamic, and adsorption isotherms were also studied. The adsorption results were fitted with various models. It was concluded that TE exhibited excellent adsorption performance for Au(III) with a maximum adsorption capacity of 256.19 mg/g. The adsorbent (TE) also showed excellent results in both selectivity and cycling performance. The study of the adsorption mechanism using XPS and DFT calculations concluded that the adsorption process is a result of the joint action of chemical and physical adsorption. In conclusion, this study presents novel methods and ideas for treating gold recovery in wastewater.

## Figures and Tables

**Table 1:** Specific surface area parameters of TE.

Category	TE
Surface area (m^2^/g)	80.1599
Average pore size (nm)	11.3548
Total pore volume (cm^3^/g)	0.262001

**Table 2:** Thermodynamic parameters.

Adsorbent	Temperature (K)	ΔG (kJ/mol)	ΔH (kJ/mol)	ΔS (kJ/mol·K)
TE	298	−18.67	24.87	0.087
	308	−19.44		
	318	−20.41		
	328	−22.58		
	338	−24.44		

**Table 3:** Parameters for the pseudo-first-order and pseudo-second-order kinetic models.

		Pseudo-First-Order Kinetic Model			Pseudo-Second-Order Kinetic Model		
Adsorbent TE		K_1_	Q_e_, cal	R_1_^2^	K_2_	Q_e_, cal	R_2_^2^
		0.03595	193.61	0.991	0.00016	235.60	0.988

**Table 4:** Langmuir isothermal model-related and Freundlich isothermal model-related parameters.

		Langmuir			Freundlich		
Adsorbent	Q_e_, exp	K_L_	Q_m_	R_1_^2^	K_F_	n	R_2_^2^
TE	256.19	2.57309	247.14	0.699	196.35724	22.75606	0.993

**Table 5:** Distribution ratio (D) and selectivity coefficient (K).

Metal Ion	Distribution Ratio (D) (mL/g)	Selectivity Coefficient (k)
Co	32.87	1497.08
Cu	136.95	359.34
Ni	31.76	1549.60
Cd	7.03	6997.04
Pb	12.68	3880.83
Zn	7.81	6304.25
Cr	781.61	62.96
Au	49,213.46	1

**Table 6:** Calculation of Fukui function for TE.

Number	Atom	N	N + 1	N − 1	f_(r)_^+^	f_(r)_^−^
1	N	−0.692	−0.726	−0.631	0.034	0.061
2	C	0.638	0.511	0.633	0.127	0.005
3	N	−0.679	−0.804	−0.437	0.125	0.242
19	N	−0.613	−0.659	0.490	0.046	1.103
21	C	−0.445	−0.435	−0.453	−0.010	−0.008

## Data Availability

The data are contained within this article.
